# Mortality during and following hospital admission among school-aged children: a cohort study

**DOI:** 10.12688/wellcomeopenres.16323.2

**Published:** 2021-01-04

**Authors:** Moses M Ngari, Christina Obiero, Martha K Mwangome, Amek Nyaguara, Neema Mturi, Sheila Murunga, Mark Otiende, Per Ole Iversen, Gregory W Fegan, Judd L Walson, James A Berkley

**Affiliations:** 1 KEMRI/Wellcome Trust Research Programme, P.O Box 230 - 80108, Kilifi, Kenya; 2 The Childhood Acute Illness & Nutrition Network (CHAIN), Nairobi, Kenya; 3 Department of Nutrition, IBM, University of Oslo, Oslo, Norway; 4 Department of Haematology, Oslo University Hospital, Oslo, Norway; 5 Division of Human Nutrition, Stellenbosch University, Tygerberg, South Africa; 6 Swansea Trials Unit, Swansea University Medical School, Swansea, UK; 7 Departments of Global Health, Medicine, Pediatrics and Epidemiology, University of Washington, Seattle, Seattle, USA; 8 Centre for Tropical Medicine & Global Health, University of Oxford, Oxford, UK

**Keywords:** Mortality, Inpatient, Post-discharge, Reason for admission, School-aged children, Cohort, Africa

## Abstract

**Background: **Far less is known about the reasons for hospitalization or mortality during and after hospitalization among school-aged children than among under-fives in low- and middle-income countries. This study aimed to describe common types of illness causing hospitalisation; inpatient mortality and post-discharge mortality among school-age children at Kilifi County Hospital (KCH), Kenya.

**Methods:** A retrospective cohort study of children 5−12 years old admitted at KCH, 2007 to 2016, and resident within the Kilifi Health Demographic Surveillance System (KHDSS). Children discharged alive were followed up for one year by quarterly census. Outcomes were inpatient and one-year post-discharge mortality.

**Results: **We included 3,907 admissions among 3,196 children with a median age of 7 years 8 months (IQR 74−116 months). Severe anaemia (792, 20%), malaria (749, 19%), sickle cell disease (408, 10%), trauma (408, 10%), and severe pneumonia (340, 8.7%) were the commonest reasons for admission. Comorbidities included 623 (16%) with severe wasting, 386 (10%) with severe stunting, 90 (2.3%) with oedematous malnutrition and 194 (5.0%) with HIV infection. 132 (3.4%) children died during hospitalisation. Inpatient death was associated with signs of disease severity, age, bacteraemia, HIV infection and severe stunting. After discharge, 89/2,997 (3.0%) children died within one year during 2,853 child-years observed (31.2 deaths [95%CI, 25.3−38.4] per 1,000 child-years). 63/89 (71%) of post-discharge deaths occurred within three months and 45% of deaths occurred outside hospital. Post-discharge mortality was positively associated with weak pulse, tachypnoea, severe anaemia, HIV infection and severe wasting and negatively associated with malaria.

**Conclusions:** Reasons for admissions are markedly different from those reported in under-fives. There was significant post-discharge mortality, suggesting hospitalisation is a marker of risk in this population. Our findings inform guideline development to include risk stratification, targeted post-discharge care and facilitate access to healthcare to improve survival in the early months post-discharge in school-aged children.

## Introduction

Despite a remarkable decline in global child mortality, more than 6 million children died in 2018, of which 0.9 million (15%) deaths occurred among children aged 5 to 14 years, mostly in low- and middle-income countries (LMICs) ^1, 2^. Public health efforts to improve survival are generally directed towards children <5 years old ^2– 4^. Despite there being no specific new health interventions targeting children aged 5 to 14 years, their mortality risk from 1990 to 2016 declined by 51% globally ^4^. However, since 2000, the annual rate of mortality reduction in this group (2.7%) has been lower than amongst children <5 years old (4.0%) ^4^.

Children ≥5 years old may be admitted to hospital with different conditions than younger children and their risks for inpatient or post-discharge mortality may also differ. However, there are only limited data describing reasons for admission and mortality-patterns in LMICs. Among school-aged children admitted to six Kenyan hospitals in 2013, 3.5% of children aged 5 to 9 years and 5.0% of children 10 to 14 years died ^5^. Infectious diseases such as malaria were the main reported causes of death in children ≥5 years ^2, 5, 6^.

Post-discharge mortality is increasingly recognized as a significant contributor to the burden of mortality among under-fives in LMICs and adults in resource-rich settings ^7– 9^. The most recent systematic review (2018) of paediatric post-discharge mortality in LMICs did not identify any studies focusing specifically on school-aged children ^7, 8^. Of 24 studies reviewed, four included children ≥5 years old. A study in Bangladesh with a sample size of 74 children aged 24 to 72 months did not report the number of children who were ≥5 years old ^10^. A large study in Kenya among children 0 to 15 years old (N=14,971) reported that 16% (88/535) of all post-hospital discharge deaths were among children aged 5 to 14 years ^11^ and two studies from Uganda with children aged 2 months to 12 years reported ~1% and 3.8% children dying after discharge, respectively, but were not disaggregated by age group ^12, 13^. Since the latest systematic review (2018) ^7^, four more studies have evaluated paediatric post-discharge mortality in LMICs ^3, 14– 16^. Hau *et al.* included children aged 2 to 12 years followed-up for 12 months after discharge from two hospitals in Tanzania and found that 16% (26/161) of children aged 5 to 12 years died after hospital discharge ^3^. Post-discharge mortality risk was reported to be higher than that of children <5 years (hazard ratio 2.44 (95%CI, 1.37 to 4.34) ^3^. The commonest diagnoses at admission were malaria and sickle cell disease ^3^. The second study from southern Mozambique included 18,023 children aged <15 years, of which 83/3,816 (2.2%) aged ≥5 years died within three months after discharge ^14^. The third study was from Kenya, but excluded children ≥5 years old ^15^. The fourth study from Tanzania included children aged 2 to 12 years, of whom 47/466 (10%) died one year after hospital discharge, but deaths were not disaggregated by age ^16^. Two of the four studies from Tanzania, used same study participants to evaluate overall one-year post-discharge mortality based on admission diagnosis ^3^ and on levels of haemoglobin ^16^ respectively.

In this retrospective cohort study, we aimed to describe the reasons for hospitalisation, underlying illnesses, and the clinical characteristics and features associated with mortality during hospitalisation and for one year after discharge among children 5 to 12 years old admitted to a rural hospital in Kenya.

## Methods

### Study setting

The study was conducted at Kilifi County Hospital (KCH), located on the Indian Ocean coast in Kenya. KCH is a secondary care hospital handling approximately 5,000 annual paediatric admissions. At KCH, approximately 60% of paediatric admissions are aged 1 to 59 months, who are mostly admitted and treated for pneumonia and diarrhoea ^15, 17, 18^. Most of the population served by the hospital are rural farmers. Clinical care at the hospital follows Kenyan and World Health Organization (WHO) guidelines.

Systematic data including clinical signs, anthropometry and laboratory investigations at admission have been collected by research clinicians and entered in a database since 1998. At discharge or death, up to two final diagnoses are assigned by the discharging clinician. From 2002, approximately 250,000 residents in an area of 891 km ^2^ neighbouring KCH have been enumerated every four months by the Kilifi Health and Demographic Surveillance System (KHDSS) for births, deaths and in- or out-migration ^19^. During each enumeration round, data collectors move from one household to another using GIS-derived maps and ask pre-specificized questions to the head of household and other members as described elsewhere ^19^. Data on admissions to KCH are linked to the KHDSS population database by matching individual child using unique ID number through predesigned database query programme. Both the KCH admissions and KHDSS databases are programmed with validation checks to improve data quality. Community enumerators within the KHDSS, clinicians and clinical assistants collecting data in the KHDSS and KCH respectively are regularly trained.

### Study population

Children aged 60 to 155 months admitted to KCH between 2007 and 2016 and resident within the KHDSS were included. The post-discharge analysis included all children discharged alive. Data from the KHDSS up to the 2018 August census round were used to confirm vital status post-discharge. 

### Study design

We performed a retrospective cohort study. Exposures evaluated were clinical and demographic features, anthropometry and laboratory variables at admission. Outcomes examined were inpatient and one-year post-discharge mortality.

### Data sources/measurement

Anthropometry, clinical history and examination, complete blood count, HIV antibody test, blood smear for malaria and blood culture were systematically conducted at admission as previously described ^20^. Anthropometry was taken by trained clinical assistants: mid-upper arm circumference (MUAC) using a non-stretchable insertion tape (TALC, St Albans, UK), weight using an electronic scale (Seca 825, Birmingham, UK) and height using a stadiometer (Seca 215, Birmingham, UK) which were regularly checked for consistency. HIV antibody testing used two rapid tests (Determine; Inverness Medical, Fl, USA; and Unigold; Trinity Biotech, Bray, Ireland). Caregivers of children with a positive HIV antibody test were counselled and referred to an HIV comprehensive care clinic. Details of subsequent outpatient clinic attendance and antiretroviral treatment were not recorded on the database. Children found to have malnutrition, sickle cell disease, tuberculosis, cardiac or neurological conditions were referred to outpatient clinics for continued care.

For this analysis, malaria was defined as a blood smear positive for *Plasmodium falciparum* and anaemia was defined as moderate (haemoglobin 8 to 11.4 g/dl) or severe (haemoglobin <8g/dl), as per WHO guidelines ^21^. Biochemical tests, radiology, sickle cell testing, lumbar puncture and other investigations were done at the discretion of the treating clinician. Meningitis was diagnosed using the cerebrospinal fluid (CSF) examination and culture as follows: positive culture for known pathogen, positive CSF microscopy (Gram stain and/or Indian ink stain), positive antigen test ( *S. pneumoniae, H. influenzae* type B, *N. meningitidis*, and *C. neoformans*) and CSF white blood cell count (WBC) ≥10 cells/µl.

### Statistical methods

Statistical analysis was performed using STATA (version 15.1; StataCorp, College Station, TX, USA). All eligible admissions and discharges from the population of KCH residents of KHDSS within the study period were included in the study, therefore, no formal sample size estimation was conducted.

MUAC-for-age z-score (MUACZ) were calculated using the method of Mramba *et al.* ^22^. Body mass index-for-age z-score (BMIZ), weight-for-age (WAZ) and height-for-age z-score (HAZ) were calculated using WHO 2007 growth references and classified as normal (≥-2Z), moderate (-3 to -2Z) and severe (<-3Z) ^23^. Anthropometric measurements and systematically collected laboratory tests were regarded as missing not at random ( *Extended data* Table S1). To ensure all children were included in the multivariable regression models, we used categorical variables and included a ‘missing’ category in the analysis.

Because the children could be admitted more than once during the study period, for the analysis of inpatient deaths we performed a multiple-admission analysis where each child could contribute more than one admission record using their unique person IDs. To examine features at admission associated with inpatient mortality, we used a backward stepwise log-binomial regression model with robust standard errors to account for multiple admissions retaining variables with a P-value <0.1 and reported adjusted risk ratios for variables with P-value <0.05 in the final multivariable model.

To calculate the post-discharge mortality rate, time at risk was defined as the date of hospital discharge until death, out-migration or 365 days later. We performed a multiple-discharge analysis where children with multiple admissions with live discharge contributed separate person-time periods when there was no overlap during the one-year follow-up. After assessing and confirming the proportional hazard assumption was not violated using the Schoenfeld residuals test, we used a Cox proportional hazard regression model with robust standard errors to account for multiple discharges to examine admission features associated with post-discharge mortality.

For both inpatient and post-discharge regression analysis, individual clinical signs, laboratory tests and final diagnoses assigned by clinical staff were used for diagnoses not captured by syndromic definitions using clinical signs. In the regression models, we used MUACZ to define undernutrition rather than BMIZ because MUACZ predicts mortality as effectively as BMIZ ^22^, is less affected by dehydration than weight-based measures ^24^, and fewer children were missing MUAC measurements. We initially excluded biochemical features that were not systematically collected and performed exploratory analyses of these features. We tested if the effects of HIV status, anaemia and malaria were modified by age and if the malaria effects on post-discharge mortality were modified by anaemia or nutritional status using likelihood-ratio tests. Goodness-of-fit of the multivariable regression models was assessed using the area under receiver operating characteristic curves (AUC) and internally validated using the bootstrapping method with 1000 resampling with replacement ^25^.

### Ethical statement

The Kenya Medical Research Institute (KEMRI) National Ethics Review Committee (SCC 2778) approved the study. Written consent for the children’s participation in the original study was provided by their parents/guardians, which included consent for subsequent analyses. 

## Results

**Figure 1.**
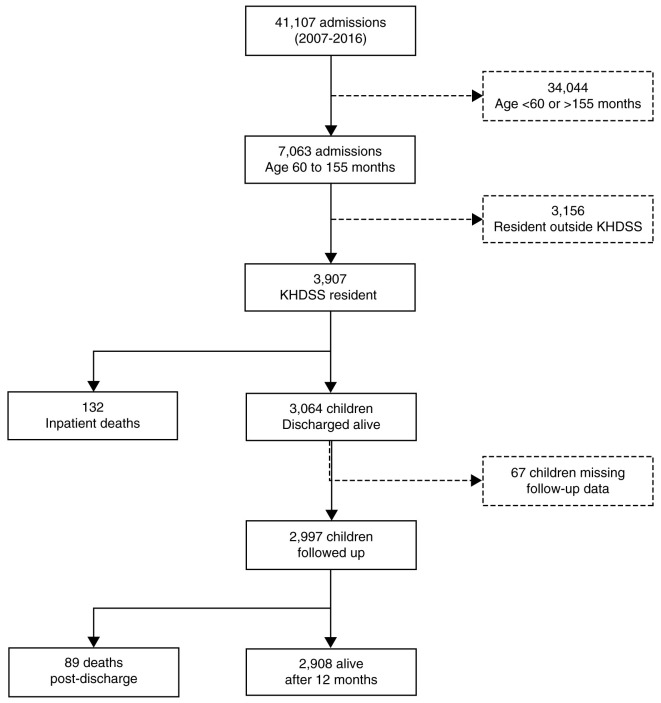
Study participant recruitment and follow-up. KHDSS, Kilifi Health Demographic Surveillance System.

From 2007 to 2016, there were 41,107 paediatric admissions to KCH, of which 7,063 (17%) were aged 5 to 12 years ( Figure 1). Of these, 3,907 (55%) admissions among 3,196 children were KHDSS residents and were included in this analysis ( Figure 1). Their median age was 92 (IQR 74−116) months and 1,673 (43%) were female. Of the 3,907 admissions, 792 (20%) presented with severe anaemia, 749 (19%) with malaria, 408 (10%) with sickle cell disease (known at admission or diagnosed during admission), 408 (10%) had suffered trauma, 340 (8.7%) with severe pneumonia and 194 (5.0%) with diarrhoea.

Underlying chronic conditions included stunting in 1,143 (29%), severe stunting in 90 (2.3%), severe wasting (MUACZ <-3) in 623 (16%), HIV infection in 194 (5.0%), and nutritional oedema in 90 (2.3%) ( Table 1, Table 2 and *Extended data* Table S2).

**Table 1.** Participants’ characteristics at admission to hospital.

Admission characteristics (N=3,907)	No. (%)
**Demographics**	
Age in months, median (IQR)	92 (74−116)
Sex (female)	1673 (43)
Had been admitted prior to age 5 years, before this study	1152 (29)
**Clinical features**	
Axillary temp <36°C	238 (6·1)
Axillary temp 36 to 37.5°C	2009 (51)
Axillary temp >37.5°C	1660 (43)
Respiratory rate <20 breaths per min	80 (2.1)
Normal respiratory rate	2179 (56)
Respiratory rate >30 breaths per min	1580 (40)
Subcostal indrawing	249 (6.4)
History of breathing difficulty	432 (11)
Hypoxia (SaO _2_ <90%)	130 (3.3)
Heart rate <80 beats per min	156 (4.0)
Normal heart rate	1694 (43)
Heart rate >120 beats per min	2035 (52)
Capillary refill >2 seconds	98 (2.5)
Temperature gradient	152 (3.9)
Weak pulse	85 (2.2)
Lethargy	417 (11)
Sunken eyes	132 (3.4)
Reduced skin turgor	71 (1.8)
Impaired consciousness ^*^	540 (14)
Severe pneumonia	340 (8.7)
Diarrhoea	194 (5.0)
**Nutritional status**	
Nutritional oedema	90 (2.3)
MUAC-for-age z-score (sd)	-1.89 (1.5)
BMI-for-age z-score (sd)	-1.37 (1.2)
Height-for-age z-score (sd)	-1.46 (1.3)
Weight-for-age z-score (sd)	-1.81 (1.2)
**Laboratory investigations**	
HIV infected	194 (5.0)
Malaria smear positive	749 (19)
Anaemia	
None (haemoglobin ≥11.5g/dl)	817 (21)
Moderate (haemoglobin 8 to 11.4g/dl)	1497 (38)
Severe (haemoglobin <8g/dl)	792 (20)
Bacteraemia	138 (3.5)
Meningitis	54 (1.4)

*Defined as presence of prostration or coma. MUAC, mid-upper arm circumference; BMI, body mass index; sd, standard deviation. 49 (1.3%) records missing MUAC, 442 (11%) records missing BMI, 287 (7.3%) records missing height-for-age z-score, 881 (23%) records missing weight-for-age z-score, 22 (0.6%) records missing heart rate, 68 (1.7%) records missing respiratory rate.

**Table 2.** Diagnoses assigned at discharge or death.

Discharge diagnosis (N=3,907)	No. (%) Diagnosis one ^*^		No. (%) Diagnosis two ^*^	
	All admissions (N=3,907)	Inpatient Deaths (N=132)	All admissions (N=3,907)	Inpatient Deaths (N=132)
Malaria	793 (20)	32 (4.0)	41 (1.1)	4 (9.8)
Sickle cell disease	408 (10)	10 (2.5)	94 (2.4)	5 (5.3)
Trauma/fractures/accidents	408 (10)	6 (1.5)	23 (0.6)	1 (4.3)
Anaemia	234 (6.0)	9 (3.8)	254 (6.5)	14 (5.5)
Epilepsy/convulsions	198 (5.1)	2 (1.0)	148 (3.8)	2 (1.4)
Lower respiratory tract infection	196 (5.0)	5 (2.6)	76 (2.0)	2 (2.6)
Snake bite	179 (4.6)	0	1 (0.03)	0
Cellulitis/pyomyositis	110 (2.8)	0	24 (0.6)	0
Gastroenteritis	110 (2.8)	0	28 (0.7)	1 (3.6)
Upper respiratory tract infection	76 (2.0)	0	38 (1.0)	0
Burns	75 (1.9)	1 (1.3)	2 (0.05)	0
Elective surgery	75 (1.9)	0	9 (0.2)	0
Malnutrition	73 (1.9)	1 (1.4)	34 (0.9)	5 (15)
Acute abdomen	69 (1.8)	3 (4.3)	4 (0.1)	0
Immunosuppression	53 (1.4)	8 (15)	114 (2.9)	12 (11)
Meningitis	48 (1.2)	12 (25)	6 (0.2)	1 (17)
Diabetes	44 (1.1)	0	3 (0.08)	0
Encephalopathy	44 (1.1)	5 (11)	11 (0.3)	3 (27)
Others	649 (17) ^‡^	38 (5.9) ^§^	225 (5.8) ^¶^	18 (8.0) ^#^
None specified	65 (1.7)	0	2,775 (71)	65 (2.3)

*Clinicians assign up to two diagnoses at death or discharge. ‡Cholera-12, rabies-9, measles-10, tetanus-15, osteomyelitis-20, asthma-44, empyema-3, pleural effusion-3, nephrotic syndrome-52, cerebral palsy-14, pyogenic arthritis-9, congenital disease-32, encephalophaty-44, hydrocephalus-5, acute flaccid paralysis-5, skin disease-19, arthritis-25, poisoning-43, unclassified disease-142, Burkitt’s lymphoma-2, dental-3, septicaemia-37, viral infection-14, urinary tract infection-22, viral hepatitis-25, pulmonary tuberculosis-20, otitis media-4, chickenpox-6, genital problems-5, conjunctivitis-5. §Immunosuppression-8, septicaemia-6, pulmonary tuberculosis-5, rabies-5, tetanus-3, heart diease-6, cerebral palsy-1, hydrocephalus-1, Burkitt’s lymphoma-1, viral hepatitis-1, chickenpox-1. ¶Cholera-4, tetanus-3, oesteomyelitis-3, asthma-15, empyema-3, pleural effusion-4, nephrotic syndrome-15, cerebral palsy-16, chromosomal abnormality-2, heart disease-13, developmental delay-5, skin disease-12, poisoning-3, unclassified disease-20, septicaemia-34, conjunctivitis-6, urinary tract infection-18, renal failure-14, viral hepatitis-8, pulmonary tuberculosis-16, otitis media-6, chickpox-3, genital problems-2, no second diagnosis-2775. #Empyema-1, cerebral palsy-1, chromosomal abnormality-1, unclassified disease-3, septicaemia-7, renal failure-3, pulmonary tuberculosis-1, chickenpox-1.

A total of 138 (3.5%) children had detectable bacteraemia ( Table 1). The commonest bacteria isolated were: *Streptococcus pneumoniae* (47/138, 34%), *Staphylococcus aureus* (32/138, 23%) and non-typhi *Salmonella species* (21/138, 15%) ( *Extended data* Table S3). The final diagnoses assigned by clinicians are shown in Table 2.

There were 132 (3.4%) inpatient deaths. The median (IQR) time to inpatient death was one (1−4) days, while median (IQR) time to discharge among the survivors was three (2−6) days. There were 44 (5.6%), 30 (4.0%), 45 (13%), 23 (17%), 9 (17%), and 10 (5.2%) deaths among those admitted with severe anaemia, malaria, severe pneumonia, bacteraemia, meningitis and diarrhoea, respectively. There were 21 (11%), 34 (5.5%), 10 (2.5%) and 4 (1.2%) deaths among children who were HIV-infected, severely wasted, had sickle cell disease and Epilepsy/convulsion, respectively ( Table 2 and *Extended data* Table S2).

### Factors associated with inpatient mortality

In the multivariable model, older age, signs of disease severity (tachypnoea, history of breathing difficulty, weak pulse and impaired consciousness), HIV infection, bacteraemia and severe stunting were positively associated with inpatient mortality, whilst epilepsy/convulsions were negatively associated with inpatient mortality ( Table 3). All variables tested in univariable analysis are shown in *Extended data* Table S4. Being severely wasted was associated with inpatient death in the univariable model, but the effect was attenuated in the multivariable model ( *Extended data* Table S4). There was no evidence that the effect of HIV infection on inpatient mortality was modified by age (P=0.13), severe wasting (P=0.97), malaria (P=0.34) or moderate and severe anaemia (P=0.13). We found no evidence that anaemia (P=0.16), age (P=0.21) or severe wasting (P=0.14) modified the effect of malaria on inpatient mortality.

**Table 3.** Multivariable regression analysis of factors associated with inpatient and post-discharge mortality.

	Inpatient analysis		Post-discharge analysis	
	Adjusted RR (95% CI)	P-value	Adjusted HR (95% CI)	P-value
Demographics				
Age in years	1.01 (1.00 to 1.02)	0.04	-	-
Clinical features at admission				
Respiratory rate per minute				
<20	2.03 (0.78 to 5.29)	0.15	- *	-
20 to 30	Reference		Reference	
>30	2.21 (1.41 to 3.47)	0.001	1.72 (1.12 to 2.63)	0.01
History of breathing difficulty	2.07 (1.40 to 3.06)	<0.001		
Weak pulse	2.18 (1.30 to 3.65)	0.003	3.54 (1.64 to 7.64)	0.001
Impaired consciousness	5.51 (3.76 to 8.10)	<0.001		
Assigned diagnosis at discharge/death				
Epilepsy/convulsions	0.30 (0.12 to 0.73)	<0.001	-	-
Investigations at admission				
HIV infected	1.75 (1.08 to 2.85)	0.02	3.06 (1.69 to 5.54)	<0.001
Malaria slide positive	-	-	0.43 (0.20 to 0.93)	0.03
Anaemia				
None	-	-	Reference	
Moderate	-	-	1.38 (0.73 to 2.61)	0.33
Severe	-	-	2.34 (1.18 to 4.63)	0.02
Bacteraemia	3.69 (2.23 to 6.10)	<0.001	-	-
Nutritional status at admission				
MUAC-for-age Z score				
≥-2	-	-	Reference	
-3 to -2	-	-	1.66 (0.95 to 2.91)	0.08
<-3	-	-	3.74 (2.24 to 6.25)	<0.001
Missing	-	-	4.99 (1.47 to 17.0)	0.01
HAZ-for-age Z score				
≥-2	Reference			
-3 to -2	0.88 (0.54 to 1.44)	0.62	-	-
<-3	2.04 (1.30 to 3.20)	0.002	-	-
Missing	1.71 (1.06 to 2.76)	0.03	-	-
Model performance				
AUC (95% CI)	0.84 (0.81 to 0.88)		0.76 (0.70 to 0.81)	
Bootstrap AUC (95% CI)	.85 (0.81 to 0.89)		0.76 (0.70 to 0.81)	

Variables are included in this table if they were selected through the backward step-wise approach and have adjusted P<0.05 for either inpatient or post-discharge analysis. All variables considered in the regression models are shown in the univariable analysis models (Table S4). MUAC, mid-upper arm circumference; HAZ, height-for-age z-score; AUC, area under receiver operating characteristics; RR, risk ratio from the log-binomial regression model; HR, hazard ratio from Cox proportion regression model, the global Schoenfeld residuals test for proportional hazard assumption P-value=0.11. *Insufficient numbers.

In exploratory analysis including biochemical variables, hyperkalaemia and elevated creatinine were associated with inpatient mortality ( *Extended data* Table S5).

### Post-discharge mortality

Among the 3,064 children who were discharged alive, follow-up data were missing for 67 (2.2%) children ( Figure 1). We therefore analysed data from 2,997 children who accrued 2,853 child-years of observation. Eighty-nine (3.0%) children died during follow-up; 63 (71%), 80 (90%) and 84 (94%) of post-discharge deaths occurred within three, six and nine months of discharge, respectively. The overall mortality rate was 31.2 (95%CI, 25.3 to 38.4) per 1,000 child-years. During the first three, six and nine months after discharge, mortality rate was 244 (95%CI, 190.3 to 311.8), 89.6 (95%CI, 72.0 to 111.6), and 53.2 (95%CI, 43.0 to 65.9) per 1000 child-years, respectively. Forty (45%) deaths occurred at home, 26 (29%) during readmission to KCH and 23 (26%) in other health facilities. Among the 26 deaths at KCH, the leading estimated causes of death were: malaria (8, 31%), anaemia (3, 12%) and heart disease (3, 12%) ( *Extended data* Table S6).

**Figure 2.**
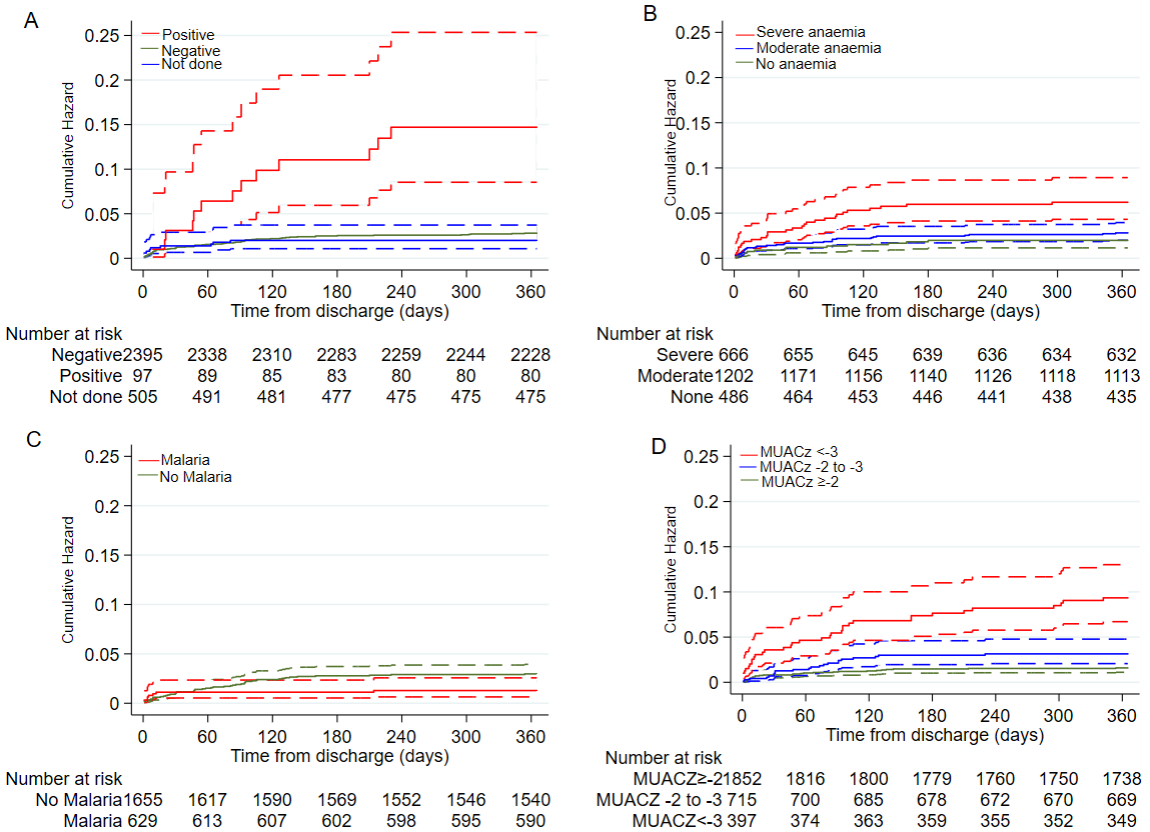
Cumulative hazard curves for post-discharge mortality: **A**) by HIV status; **B**) by anaemia categories; **C**) by malaria status and **D**) by mid-upper arm circumference (MUAC)-for-age z-scores groups. The broken lines are 95% confidence intervals, where there is overlap in 95%CI some lower or upper confidence intervals may not be visible.

Some signs of disease severity at admission (elevated respiratory rate and presence of weak pulse), HIV infection, severe anaemia and severe wasting were positively associated with post-discharge death ( Table 3 and Figure 2). Malaria was negatively associated with post-discharge death ( Table 3 and Figure 2). Hospital admission duration was not independently associated with post-discharge death ( *Extended data* Table S4). Thirteen (0.43%) children absconded from hospital and all of them were alive after one year post-discharge. 

There was no evidence that the effect of HIV infection on post-discharge mortality was modified by age (P=0.95), anaemia (P=0.25), malaria (P=0.37) or severe wasting (P=0.88). The effect of malaria on post-discharge mortality was not modified by anaemia (P=0.33) but was modified by severe wasting (P=0.007) and weak evidence of being modified by age (P=0.05). Compared to MUACZ ≥-2 with a negative malaria slide, the groups of MUACZ -3 to -2 with a negative malaria slide (HR 3.60 [95%CI, 1.75 to 7.39]) and MUACz<-3 with a negative malaria slide (HR 6.36 [95%CI, 3.10 to 13.1]) had greater post-discharge mortality. Among children with positive slide results, MUACZ was not associated with post-discharge mortality.

In the exploratory analysis of biochemical variables, hyperkalaemia and elevated creatinine were associated with post-discharge mortality ( *Extended data* Table S7).

## Discussion

In this large study of school-aged children admitted to hospital and systematically followed-up during admission and for one year after discharge, we observed that anaemia, malaria, sickle cell disease and trauma were the leading reasons for admission. This profile is unlike that reported among under-fives, in whom pneumonia and diarrhoea are the leading reasons for admission, accounting for more than two-thirds of hospital admission in Kenya and South Africa ^26– 28^. All-cause inpatient case fatality was 3.4%, which was similar to the 3.5% previously reported in six hospitals in Kenya among children 5 to 17 years old in 2013 ^5^. Markers of disease severity at admission were the main predictors of inpatient mortality despite following WHO care guidelines, suggesting that current management strategies and resources may be insufficient for the sickest school-aged children. Surprisingly, epilepsy/convulsion appeared ‘protective’ compared to other reasons for admission in this hospitalised population, but would not be expected to be protective if compared to children in the community.

 The 3.0% one-year post-discharge mortality, with almost three-quarters occurring within three months, broadly concurs with that observed in rural Mozambique three months after hospital-discharge (2.2%) among children 5 to 15 years old ^14^, but was much lower than one-year post-discharge mortality (16%) observed among children 5 to 12 years old in Tanzania ^3^. However, the Tanzanian study did not stratify results by age above or below 5 years and it seems likely that a higher prevalence of non-communicable diseases including cancer and heart disease reported in that population may have contributed to the higher risk of post-discharge death. However, the mortality rate of 31.2 deaths/1000 child-years observed was >34 fold higher than 0.91 deaths/1000 child-years within KHDSS among this age-group (unpublished data). The leading cause of death among the 26 children who died during readmission at KCH was malaria, similar to the leading cause assigned through verbal autopsies to children 1 to 4 years old who died in the community, but differing from that in infants among whom pneumonia was the leading causes of death in KHDSS ^29^

Tachypnoea, which may be associated with anaemia, hypoxia, sepsis or pneumonia, and weak pulse, a sign of circulatory insufficiency, were independently associated with post-discharge mortality which would suggest some children may be discharged with ongoing unstable vital signs that may lead to post-discharge deaths. Undernutrition was associated with post-discharge mortality, similar to most previous studies, which have identified nutritional status as a major risk-factor for post-discharge mortality in under-fives ^3, 7, 14, 15, 17, 30^. We previously showed that MUACZ is valuable in predicting post-discharge mortality among children >5 years in a model only adjusted for age, sex and HIV status ^22^.

We found severe anaemia was independently associated with post-discharge mortality; however, prior evidence of the effect of haemoglobin concentration on post-discharge mortality has been inconsistent, and may be influenced by the predominant causes of anaemia and local transfusion policies ^3, 14, 16, 31– 34^. Interestingly, we found no significant effect modification by anaemia on the effect of malaria on post-discharge mortality. The finding that children with malaria had lower post-discharge mortality than other reasons for admission is consistent with previous reports from this site among children <5 years of age where malaria parasitaemia had an apparently ‘protective’ effect on post-discharge mortality ^11^. This likely reflects the fact that when appropriately treated, mortality risk in children with malaria may be lower than in children with other causes of a similar apparent severity of illness.

In exploratory analyses, elevated creatine and hyperkalaemia, markers of impaired kidney function, were identified as predictors of both inpatient and post-discharge mortality. One study among children aged 2 to 12 years in Tanzania identified estimated glomerular filtration rate <60 ml/min/1·73m ^2^ as a predictor of one-year post-discharge mortality ^3^. This is an important finding since dehydration and sepsis are common and associated with acute kidney injury ^35^, and current guidelines recommend potentially nephrotoxic empiric gentamicin, without capacity for monitoring levels ^36^. However, clinical trials that could delineate attributable nephrotoxicity from background risks from renal disease, serious illness and dehydration have not yet been done.

Overall, our findings add to previous data in under-fives suggesting that hospitalisation marks an extended period of vulnerability, and reports suggesting that significant post-discharge mortality occurs outside hospital even among school-age children ^7, 11, 17^. The transition from inpatient to home care in the immediate three-month period following hospital discharge appears to be a critical period of risk. Risk stratification and targeted interventions for this population during this period might improve survival ^4^. However, current services, such as for the management of malnutrition in the community or those to manage anaemia, largely focus on children <5 years of age and may not operate in a way that fully addresses the mortality risk in this population ^37, 38^.

Strengths of this study include the large sample size, high rates of follow-up and the detailed longitudinal data available. However, this study also had some limitations. We used data from a single hospital, including only children resident within the nearby KHDSS, thus our results may not be generalizable to other sites or children living further away from the main road. Biochemical features were not systematically collected and could not be included in the primary analysis. We were not able to ascertain whether HIV infected children and malnourished children attended and complied with treatments from comprehensive care and nutrition clinics after discharge from hospital. We did not have access to causes of deaths among children who died at home or in other health facilities. Moreover, this study did not include data on caregiver or household characteristics, socio-economic situation or access to care.

## Conclusion

This study highlights important differences in reasons for hospitalisation among school-aged children compared to younger children, and high rates of inpatient and post-discharge mortality among subgroups of school-aged children. To improve survival, active risk stratification and targeting intervention and follow-up in the early months after hospital discharge are needed, along with expansion of services that normally focus on the under-fives to vulnerable over-fives. The large proportion of deaths outside hospital suggests that facilitating access to healthcare among the most vulnerable, and priority clinical evaluation if unwell may be important measures. 

## Data availability

### Underlying data

Harvard Dataverse: Replication Data for: Inpatient and post-discharge mortality among children 5-12 years old in rural Kenya. https://doi.org/10.7910/DVN/ZJOUWB ^39^.

The project contains the following underlying data:

- Over5years_multipleadmissions.csv (contains clinical, anthropometric, CBC, blood & CSF culture at the time of hospital admission for children aged 5 to 12 years from 2007 to 2016, also provided in .dta format).
- over5years_khdss.csv (contains vital status in the community following hospital discharge, also provided in .dta format).
- over5yearschemistry.csv (contains the biochemistry variables that were not systematically collected at admission. This file is used to run a sub-analysis of the chemistry factors associated with both inpatient and post-discharge mortality, also provided in .dta format).
- Data_Dictionary_NgariMM.pdf (contains a list of the variables of data collected at admission and discharge and their description).
- Discharge_diagnosis codes.csv (contain the list of codes for discharge diagnosis).

### Extended data

Harvard Dataverse: Replication Data for: Inpatient and post-discharge mortality among children 5-12 years old in rural Kenya. https://doi.org/10.7910/DVN/ZJOUWB ^39^.

This project contains the following extended data:

- Additional data_Mngari et.al 2020.pdf (contains supplementary Tables S1−S5).
- Data_Readme_NgariMM.txt (dataset description and usage instructions).
- 5older years analysis_v1.do (STATA script used to generate the summary participants characteristics at admission, reasons for admission to hospital and inpatient mortality including factors associated with inpatient deaths).
- post-discharge analysis_over5years.do (STATA script that runs the post-discharge analysis. It merges the Over5years_multipleadmissions.dta with the over5years_khdss.dta, computes time under follow-up, post-discharge deaths, mortality rates and factors associated with post-discharge deaths).

### Reporting guidelines

Harvard Dataverse: STROBE checklist for “Replication Data for: Inpatient and post-discharge mortality among children 5-12 years old in rural Kenya”. https://doi.org/10.7910/DVN/ZJOUWB ^39^.

Data are available under the terms of the Creative Commons Attribution 4.0 International (CC BY 4.0 )
