# Supplementary material for: Mortality during and following hospital admission among school-aged children: a cohort study
Source: Wellcome Open Res. 2021 Jan 4;5:234. Originally published 2020 Oct 8. [Version 2] doi: 10.12688/wellcomeopenres.16323.2 (PMC7656274; doi:10.12688/wellcomeopenres.16323.2)
Supplement: Peer review history

# Peer review: Reviewer response for version 2

**DOI:** 10.21956/wellcomeopenres.18189.r41934

**Published:** 2021-01-08

**Authors:** Bruno Masquelier

**Affiliations:** 1 Demography Research Center, Catholic University of Louvain, Leuven, Belgium

All comments have been addressed. Thank you.

Is the work clearly and accurately presented and does it cite the current literature?

Yes

If applicable, is the statistical analysis and its interpretation appropriate?

Yes

Are all the source data underlying the results available to ensure full reproducibility?

Yes

Is the study design appropriate and is the work technically sound?

Yes

Are the conclusions drawn adequately supported by the results?

Partly

Are sufficient details of methods and analysis provided to allow replication by others?

Yes

Reviewer Expertise:

Demography.

I confirm that I have read this submission and believe that I have an appropriate level of expertise to confirm that it is of an acceptable scientific standard.

# Peer review: Reviewer response for version 2

**DOI:** 10.21956/wellcomeopenres.18189.r41932

**Published:** 2021-01-05

**Authors:** Matthew Wiens

**Affiliations:** 1 University of British Columbia, Vancouver, Canada

No new comments.

Is the work clearly and accurately presented and does it cite the current literature?

Yes

If applicable, is the statistical analysis and its interpretation appropriate?

Yes

Are all the source data underlying the results available to ensure full reproducibility?

Yes

Is the study design appropriate and is the work technically sound?

Yes

Are the conclusions drawn adequately supported by the results?

Yes

Are sufficient details of methods and analysis provided to allow replication by others?

Yes

Reviewer Expertise:

Post-discharge outcomes research, Epidemiology, Prediction modelling.

I confirm that I have read this submission and believe that I have an appropriate level of expertise to confirm that it is of an acceptable scientific standard.

# Peer review: Reviewer response for version 1

**DOI:** 10.21956/wellcomeopenres.17944.r40989

**Published:** 2020-11-10

**Authors:** Matthew Wiens

**Affiliations:** 1 University of British Columbia, Vancouver, Canada

This is another excellent study by the Kilifi team and their colleagues which further highlights the importance of pediatric post-discharge mortality in resource limited settings, this time among a new population of children that has to date been largely neglected - school age children. I applaud this group for this new focus and believe that as evidence such as this continues to accumulate, that policy makers and others involved in the development and implementation of guidelines and recommendations, will increasingly see the provision of improved discharge and post-discharge care as a major priority to further improve child health outcomes. 

 This study is well justified and the reasons are well outlined in the introduction. The methodology follows a similar strategy as other previously published work by this team and is clear and concisely recorded.

 Methods:

 The methodology follows a similar strategy as other previously published work by this team and is clear and concisely recorded. Given that this study is retrospective in nature, and utilized data routinely collected by health workers during routine care, some mention of data quality should be considered, both in terms of standardized training, as well as quality assessment. 

 In terms of missing data, I see some minor statements at the bottom of Table 1, but this does not comprehensively state missingness in its totality. Would it perhaps be better to have another column in this table outlining the % missing for each for the variables of potential interest? For example, how many were missing "clinical features", how many were missing temp, how many were missing a diagnosis, etc. I recognize that some variables are defined by their presence (lethargy, decreased skin turger, etc.), thus missingness is impossible to determine, but for those that are not subject to this issue, a report of missingness will give the readers an overall picture of the quality of the data from which these analyses are conducted. I do note that nearly 1 in 4 are missing WAZ, which does seem quite high.

 For children who were below 155 months, but would be older than 155 months prior to the completion of 12 month follow-up, did these children remain in the cohort beyond the 155 month upper age limit? If yes, did the KHDSS properly capture outcomes among these older children?

 Results:

 The results are adequately presented given the analyses conducted. Perhaps more details could be presented on missingness in the tables.

 Did the authors examine discharge disposition as an associated factor with death (i.e. discharge against medical advice).

 I note that the authors mention that 45% of deaths did not occur during readmission to KCH, but does this also mean that they died outside of ANY health facility? 

 For those that died during a readmission, can these causes of death be briefly described?

 Was any comparative exploration done on early vs late post-discharge deaths?

 Was any exploration on readmissions in general done?

 Discussion

 The discussion touches on the main points needed for this topical area of research. I have no further comments.

Is the work clearly and accurately presented and does it cite the current literature?

Yes

If applicable, is the statistical analysis and its interpretation appropriate?

Yes

Are all the source data underlying the results available to ensure full reproducibility?

Yes

Is the study design appropriate and is the work technically sound?

Yes

Are the conclusions drawn adequately supported by the results?

Yes

Are sufficient details of methods and analysis provided to allow replication by others?

Yes

Reviewer Expertise:

Post-discharge outcomes research, Epidemiology, Prediction modelling.

I confirm that I have read this submission and believe that I have an appropriate level of expertise to confirm that it is of an acceptable scientific standard.

# Response

**Published:** 2020-12-10

**Authors:** Moses Ngari

**Affiliations:** Kenya Medical Research Institute, Kenya

We thank the reviewer for his comments. This has provided an opportunity to improve the manuscript. Below find point-by-point responses in italics:

 This study is well justified and the reasons are well outlined in the introduction. The methodology follows a similar strategy as other previously published work by this team and is clear and concisely recorded.

*Thank for the compliment.*

 Methods:

 The methodology follows a similar strategy as other previously published work by this team and is clear and concisely recorded. Given that this study is retrospective in nature, and utilized data routinely collected by health workers during routine care, some mention of data quality should be considered, both in terms of standardized training, as well as quality assessment. 

*We added details about training, validation checks in the databases and measurements equipment consistency checks in the methods (study settings and Data sources/measurement)*

 In terms of missing data, I see some minor statements at the bottom of Table 1, but this does not comprehensively state missingness in its totality. Would it perhaps be better to have another column in this table outlining the % missing for each for the variables of potential interest? For example, how many were missing "clinical features", how many were missing temp, how many were missing a diagnosis, etc. I recognize that some variables are defined by their presence (lethargy, decreased skin turger, etc.), thus missingness is impossible to determine, but for those that are not subject to this issue, a report of missingness will give the readers an overall picture of the quality of the data from which these analyses are conducted. I do note that nearly 1 in 4 are missing WAZ, which does seem quite high.

*We already had a table of numbers missing anthropometry (Extended data Table S1). Approximately 23% of children were missing WAZ because it is incalculable for children >120 months old. WAZ has not been used in the regression analysis. We have added a new table (Extended data Table s1) with a list of variables with missing values and explained under statistical methods that anthropometric measurements and systematically collected laboratory tests were regarded as missing not at random and a ‘missing’ category was added in the regression models.*

 For children who were below 155 months, but would be older than 155 months prior to the completion of 12 month follow-up, did these children remain in the cohort beyond the 155 month upper age limit? If yes, did the KHDSS properly capture outcomes among these older children?

*Yes, a child older than 155 after 12 months follow-up would still remain in study. The KHDSS census enumerates the entire population including adults. So the one-year outcome within the KHDSS was properly captured.*

   

 Results:

 The results are adequately presented given the analyses conducted. Perhaps more details could be presented on missingness in the tables.

*We have added an extra table with list of variables with missing values (Extended data Table s1)*

 Did the authors examine discharge disposition as an associated factor with death (i.e. discharge against medical advice).

*We have added a sentence in the results section (under post-discharge mortality section). 13 (0.43%) children absconded from hospital and all of them were alive after one-year post-discharge.*

 I note that the authors mention that 45% of deaths did not occur during readmission to KCH, but does this also mean that they died outside of ANY health facility? 

*We have provided more details in the results section. Briefly, 40 deaths occurred at home, 26 during re-admission at KCH and 23 in other health facilities.*

 For those that died during a readmission, can these causes of death be briefly described?

*Yes, we have provided the estimated causes if death for the 26 deaths during readmission at KCH (Extended data Table S6).*

 Was any comparative exploration done on early vs late post-discharge deaths?

*No. We did not consider any exploration because of the few post-discharge deaths (63 Vs 26 deaths).*

 Was any exploration on readmissions in general done?

*No. Our pre-specified endpoints were inpatient and post-discharge deaths. Hospital readmission can be examined as a separate publication.*

   

 Discussion

 The discussion touches on the main points needed for this topical area of research. I have no further comments.

*Thank you.*

# Peer review: Reviewer response for version 1

**DOI:** 10.21956/wellcomeopenres.17944.r41033

**Published:** 2020-11-06

**Authors:** Duncan Hau

**Affiliations:** 1 Weill Cornell Medical College, New York, NY, USA

This large retrospective cohort study is of children 5-12 years of age admitted to a hospital. The authors evaluated causes of admissions and mortality of in-hospital/post-hospital up to 1 year. This study adds valuable information to the current research on post-discharge mortality, particularly for children over five years. The strengths of this study are the large participates and high follow-up rate. The authors have done a very good job presenting the data in a clear and concise form. I recommend this manuscript for indexing with a few minor revisions listed below. 

 Introduction:

- The authors do a thorough review of the current literature on post-hospital mortality and state the problem well. 
- In Paragraph #3, the authors mention since the last review (2018), four more studies have evaluated pediatric post-discharge mortality. Reference 3 and 16 look to be from the same study subjects in Tanzania. Ref 16 evaluated the subjects based on level of hemoglobin compared to ref 3 that evaluated on diagnoses. Might want to clarify this in the text. 
- Aim of study is clear. I would recommend mentioning what type of study in the last paragraph (i.e.: retrospective cohort study). 

Methods:

- Methods are well written with clear study design and statistical analysis used.
- The authors mention the use of KHDSS for post-hospital period to confirm vital status post-discharge. It would be good to mention briefly how families are contacted by the KHDSS, and if children die outside the health care system (i.e.: at home), was there a way to determine cause of death? 

Results:

- The results are clearly presented in text and tables/figures. 
- It is interesting to note the in-patient deaths vary considerably by diagnosis (i.e.: severe pna, bacteremia, meningitis have mortality 13-17% vs. severe anaemia and malaria 4-5%). Do you know the causes of death for the post-hospital children?
- Table 3: It is interesting that epilepsy/convulsions was found to be protective for inpatient mortality. Any thoughts to elaborate this finding in the discussion?
- Post-hospital mortality 3.0% with majority of deaths (71%) within the first 3 months indicate this is a critical period for children. 
- It is interesting to note that some signs at admission you found in your study (i.e.: elevated RR, weak pulse) were associated with post-discharge death. These were also variables that were associated with in-patient death. The assumption that children are discharged from the hospital with stable vital signs (normal RR and normal pulse strength) would make me think these variables (unstable vital signs or concerning physical examination findings) would only be factors for in-patient mortality. I think in resource limited settings, some children are perhaps discharged with unstable vital signs which can lead to death post-discharge. 
- The authors mentioned 45% deaths occurred without readmission to KCH. Could these children have been admitted to another health care facility in the area? I don’t think we can assume they died “outside hospital” unless that was verified. This would lead back to explaining in the methods section how families were contacted post-discharge and what questions were asked.
- Do you know causes of death for the post-hospital mortality? If yes, it would be nice to know and if these differ from the admission diagnosis. For example, a child admitted for malaria was discharged and then died post-hospital due to another cause. 

Discussion: 

- In paragraph #6 about exploratory analyses, the authors mention ref #3 for a study that identified GFR. The authors wrote “2 to 15 years”, but it appears that study evaluated 2-12 year olds. This should be corrected in the text. 
- In paragraph #7 when you state “our findings add to previous data in under-fives suggesting..”, shouldn’t it be “over-five” since your study is on school aged children?

Is the work clearly and accurately presented and does it cite the current literature?

Yes

If applicable, is the statistical analysis and its interpretation appropriate?

Yes

Are all the source data underlying the results available to ensure full reproducibility?

Yes

Is the study design appropriate and is the work technically sound?

Yes

Are the conclusions drawn adequately supported by the results?

Yes

Are sufficient details of methods and analysis provided to allow replication by others?

Yes

Reviewer Expertise:

Pediatric Hospital Outcomes Research.

I confirm that I have read this submission and believe that I have an appropriate level of expertise to confirm that it is of an acceptable scientific standard.

# Response

**Published:** 2020-12-10

**Authors:** Moses Ngari

**Affiliations:** Kenya Medical Research Institute, Kenya

We thank the reviewer for his comments. This has provided an opportunity to improve the manuscript. Below find point-by-point responses in italics:

 Introduction:

- The authors do a thorough review of the current literature on post-hospital mortality and state the problem well.

*Thank you for the compliment.*

  

- In Paragraph #3, the authors mention since the last review (2018), four more studies have evaluated pediatric post-discharge mortality. Reference 3 and 16 look to be from the same study subjects in Tanzania. Ref 16 evaluated the subjects based on level of hemoglobin compared to ref 3 that evaluated on diagnoses. Might want to clarify this in the text

*Thank you for pointing out that the two studies used the same study participants. We have highlighted this in the introduction (last sentence of paragraph 3)*

  

- Aim of study is clear. I would recommend mentioning what type of study in the last paragraph (i.e.: retrospective cohort study). 

*We have added the type of study in the last paragraph of background section.*

 Methods:

- Methods are well written with clear study design and statistical analysis used.

*Thank you for the compliment.* 

  

- The authors mention the use of KHDSS for post-hospital period to confirm vital status post-discharge. It would be good to mention briefly how families are contacted by the KHDSS, and if children die outside the health care system (i.e.: at home), was there a way to determine cause of death? 

*We added some description of KHDSS in the study setting section. In the results we have provided a breakdown of the location of death for the 89 post-discharge deaths. For the deaths at home or in other health facilities, we do not have access to the causes of death. We have added this as a study limitation in the last paragraph of discussion. For the 26 deaths that occurred during readmission at KCH we have added the estimated causes of deaths (Extended data Table S6).*

 Results:

- The results are clearly presented in text and tables/figures.

*Thank you for the compliment.*

  

- It is interesting to note the in-patient deaths vary considerably by diagnosis (i.e.: severe pna, bacteremia, meningitis have mortality 13-17% vs. severe anaemia and malaria 4-5%). Do you know the causes of death for the post-hospital children?

*As mentioned in previous response, we have no access to causes of deaths for deaths that occurred at home or in other health facilities. However, for the deaths that occurred at KCH, we have provided the causes in the results section.*

  

- Table 3: It is interesting that epilepsy/convulsions was found to be protective for inpatient mortality. Any thoughts to elaborate this finding in the discussion?

*Epilepsy/convulsions is only ‘protective’ compared to other reasons for admission. It would not be protective if comparing to the community. We have added a sentence to clarify this in the first paragraph of discussion.*

  

- Post-hospital mortality 3.0% with majority of deaths (71%) within the first 3 months indicate this is a critical period for children. 

*Yes, the transition from hospital to home is a critical period for this group of children. We have this highlighted on paragraph 6 of discussion.*

- It is interesting to note that some signs at admission you found in your study (i.e.: elevated RR, weak pulse) were associated with post-discharge death. These were also variables that were associated with in-patient death. The assumption that children are discharged from the hospital with stable vital signs (normal RR and normal pulse strength) would make me think these variables (unstable vital signs or concerning physical examination findings) would only be factors for in-patient mortality. I think in resource limited settings, some children are perhaps discharged with unstable vital signs which can lead to death post-discharge.

 

*We agree, it is possible that children are discharged with unstable vital signs. However, most studies have no access to discharge clinical signs and thus not able to evaluate their effects on post-discharge outcomes. We have also highlighted this is in paragraph 3 of discussion.*

  

- The authors mentioned 45% deaths occurred without readmission to KCH. Could these children have been admitted to another health care facility in the area? I don’t think we can assume they died “outside hospital” unless that was verified. This would lead back to explaining in the methods section how families were contacted post-discharge and what questions were asked.

*We have responded in the previous comment. Briefly all the 40 (45%) occurred at home with another 23 (26%) occurring in other health facilities.*

 ·  Do you know causes of death for the post-hospital mortality? If yes, it would be nice to know and if these differ from the admission diagnosis. For example, a child admitted for malaria was discharged and then died post-hospital due to another cause.

*We don’t have access to causes of deaths for those occurring at home or in other health facilities (60, 71%) deaths. We added a table (Extended data Table S6) showing causes of deaths for the 26 deaths that occurred during readmission at KCH. Looking at the frequencies of the estimated causes of deaths among the 26 deaths at KCH, it is likely they correlate with admission/discharge diagnosis (Malaria (8 deaths) and anaemia (3deaths) were the leading causes of deaths which were also leading causes of admission/discharge diagnosis). However, because of few numbers of causes of deaths available (29%) and possible selection bias of only including these deaths, we have not explored formal testing for diagnosis correlations.*

 Discussion: 

- In paragraph #6 about exploratory analyses, the authors mention ref #3 for a study that identified GFR. The authors wrote “2 to 15 years”, but it appears that study evaluated 2-12 year olds. This should be corrected in the text. 

*Thank you for picking this, we have corrected.*

  

- In paragraph #7 when you state “our findings add to previous data in under-fives suggesting..”, shouldn’t it be “over-five” since your study is on school aged children?

*As we mentioned in the background, we found no study reporting post-discharge mortality specifically on children over-five years. Therefore, the study findings extend what has been observed in under-fives. We have made the statement clearer in the discussion (paragraph 6).*

# Peer review: Reviewer response for version 1

**DOI:** 10.21956/wellcomeopenres.17944.r40793

**Published:** 2020-10-23

**Authors:** Bruno Masquelier

**Affiliations:** 1 Demography Research Center, Catholic University of Louvain, Leuven, Belgium

This article provides detailed and very useful information for the prevention of deaths in school-age children, showing that the experience of a hospital stay is an important marker of excess mortality in the future. I only have minor comments.

- The authors report that 3% of children died within one year period. But what is the baseline mortality rate in this age group in the Kilifi HDSS?
- The study is located within the KHDSS site. It would be nice to add some background information in the intro or discussion about the causes of death in children aged 5-12 from the verbal autopsies to compare with causes of post-discharge deaths reported in the study. 
- In the methods section, it is said that details of subsequent clinic attendance were not recorded on the database. This is a bit confusing as on page 4, the authors acknowledge that children may be admitted multiple times and each child could contribute more than one admission. Can you clarify? Are you referring to the lack of data on subsequent clinic attendance when children are admitted in other hospitals (possibly outside the HDSS) after being discharged from KCH? 
- One important result is that 45% of deaths occurred without readmission to KCH. Among these, some children could have been admitted in other hospitals or in health facilities (and die before being referred to KCH). Based on info from the HDSS (e.g. verbal autopsies), is it possible to know the share of deaths that occurred outside of health facilities?    
- Can you add more details about how the patient data are linked to the HDSS database? Is this record linkage deterministic based on some kind of ID number or performed on-site through searches in the HDSS database?
- The abstract states that reasons for admissions were markedly different from those reported in under-fives, but what are these? These are only mentioned very quickly in the discussion, with only two reasons (pneumonia and diarrhoea). More details should be provided on these, ideally from KCH.   
- Second paragraph of the main text, "children may be admitted", please specify in the hospital or/health facilities. 
- Page 3, "5 to 12" instead of '5 to12'.
- In Table 1, I don't understand why 29% of children of the sample of 3907 children were admitted prior to age 5 years as these 3907 admissions are supposed to be of children aged 5-12 years (from the description of the sample in the Results section). Can you clarify?
- In Figure 2, can you add confidence intervals around the cumulative hazard curves?

Is the work clearly and accurately presented and does it cite the current literature?

Yes

If applicable, is the statistical analysis and its interpretation appropriate?

Yes

Are all the source data underlying the results available to ensure full reproducibility?

Yes

Is the study design appropriate and is the work technically sound?

Yes

Are the conclusions drawn adequately supported by the results?

Partly

Are sufficient details of methods and analysis provided to allow replication by others?

Yes

Reviewer Expertise:

Demography.

I confirm that I have read this submission and believe that I have an appropriate level of expertise to confirm that it is of an acceptable scientific standard, however I have significant reservations, as outlined above.

# Response

**Published:** 2020-12-10

**Authors:** Moses Ngari

**Affiliations:** Kenya Medical Research Institute, Kenya

We thank the reviewer for their comments. This has provided an opportunity to improve the manuscript. Below find point-by-point responses in italics:

 This article provides detailed and very useful information for the prevention of deaths in schoolmate

 children, showing that the experience of a hospital stay is an important marker of excess mortality in the future. I only have minor comments.

*Thank you for the compliment.*

 The authors report that 3% of children died within one year period. But what is the baseline

 mortality rate in this age group in the Kilifi HDSS?

*The baseline mortality rate in this age group within KHDSS is **0.91 deaths per 1000 PYO **(Unpublished). Therefore, given our post-discharge mortality rate of 31.2 deaths per 1000 PYO, children were 34 times more likely to died compared to their community peers. We have added this in the discussion (second paragraph).*

 ○ The study is located within the KHDSS site. It would be nice to add some background

 information in the intro or discussion about the causes of death in children aged 5-12 from

 the verbal autopsies to compare with causes of post-discharge deaths reported in the

 study.

*We have added some description of the causes of deaths within KHDSS assigned through verbal autopsies and a reference ( https://pubmed.ncbi.nlm.nih.gov/25377342/) in the discussion (second paragraph).*

 ○ In the methods section, it is said that details of subsequent clinic attendance were not

 recorded on the database. This is a bit confusing as on page 4, the authors acknowledge

 that children may be admitted multiple times and each child could contribute more than

 one admission. Can you clarify? Are you referring to the lack of data on subsequent clinic

 attendance when children are admitted in other hospitals (possibly outside the HDSS) after

 being discharged from KCH?

*Thank for the question. We are referring to subsequent outpatient clinic attendance for management of chronic illness like HIV and TB. We have added the word “outpatient” to make the sentence clearer*. 

 ○ One important result is that 45% of deaths occurred without readmission to KCH. Among

 these, some children could have been admitted in other hospitals or in health facilities (and

 die before being referred to KCH). Based on info from the HDSS (e.g. verbal autopsies), is it

 possible to know the share of deaths that occurred outside of health facilities?

*Yes, we have added these numbers in the results section. In brief; 40 (45%) deaths occurred at home, 26 (29%) occurred during readmission at KCH and 23 (26%) occurred in other health facilities. In the discussion we also discussed some possible causes of deaths in this age-group assigned by verbal autopsies from previous publication ( https://pubmed.ncbi.nlm.nih.gov/25377342/).*

 ○ Can you add more details about how the patient data are linked to the HDSS database? Is

 this record linkage deterministic based on some kind of ID number or performed on-site

 through searches in the HDSS database?

*We have added more details under the study settings in the methods section*.

 ○ The abstract states that reasons for admissions were markedly different from those

 reported in under-fives, but what are these? These are only mentioned very quickly in the

 discussion, with only two reasons (pneumonia and diarrhoea). More details should be

 provided on these, ideally from KCH.

*We added a sentence in the methods section: study settings to the effects that ~60% of admissions at KCH are children 1 to 59 months old and majority are admitted with pneumonia and diarrhoea and provided references.  * 

 ○ Second paragraph of the main text, "children may be admitted", please specify in the

 hospital or/health facilities.

*We have added the missing words “to hospital”*

 ○ Page 3, "5 to 12" instead of '5 to12'.

*Thank you, we have corrected.*

 ○ In Table 1, I don't understand why 29% of children of the sample of 3907 children were

 admitted prior to age 5 years as these 3907 admissions are supposed to be of children aged

 5-12 years (from the description of the sample in the Results section). Can you clarify?

*Thank for the comment, we understand this could be confusing. We have clarified in Table 1 “Had been admitted prior to age 5 years before this study”*

 ○ In Figure 2, can you add confidence intervals around the cumulative hazard curves?

*We have added 95% CI around the cumulative hazard curves as recommended (new Fig 2).*
